# The genome sequence of the Muslin moth, *Diaphora mendica *(Clerck, 1759)

**DOI:** 10.12688/wellcomeopenres.19540.1

**Published:** 2023-06-21

**Authors:** Douglas Boyes, Peter W.H. Holland, University of Oxford and Wytham Woods Genome Acquisition Lab, Darwin Tree of Life Barcoding collective, Wellcome Sanger Institute Tree of Life programme, Wellcome Sanger Institute Scientific Operations: DNA Pipelines collective, Tree of Life Core Informatics collective, Darwin Tree of Life Consortium

**Affiliations:** 1 UK Centre for Ecology & Hydrology, Wallingford, England, UK; 2 University of Oxford, Oxford, England, UK

**Keywords:** Diaphora mendica, the Muslin moth, genome sequence, chromosomal, Lepidoptera

## Abstract

We present a genome assembly from an individual male *Diaphora mendica* (the Muslin moth; Arthropoda; Insecta; Lepidoptera; Erebidae). The genome sequence is 748.7 megabases in span. Most of the assembly is scaffolded into 26 chromosomal pseudomolecules, including the Z sex chromosome. The mitochondrial genome has also been assembled and is 15.41 kilobases in length.

## Species taxonomy

Eukaryota; Metazoa; Eumetazoa; Bilateria; Protostomia; Ecdysozoa; Panarthropoda; Arthropoda; Mandibulata; Pancrustacea; Hexapoda; Insecta; Dicondylia; Pterygota; Neoptera; Endopterygota; Amphiesmenoptera; Lepidoptera; Glossata; Neolepidoptera; Heteroneura; Ditrysia; Obtectomera; Noctuoidea; Erebidae; Arctiinae; Spilosomini; *Diaphora*; *Diaphora mendica* (Clerck, 1759) (NCBI:txid987922).

## Background

Lepidoptera generally show limited sexual dimorphism in wing patterning: in most species males and females have similar markings and colouration. Exceptions include the Orange-tip butterfly *Anthocaris cardamines*, with orange wingtips only in males, and the Emperor moth *Saturnia pavonia* with major size and colour differences between sexes. The Muslin moth *Diaphora mendica* is an interesting case, as it has conspicuous wing colour difference between sexes plus colour polymorphism in males only.

*D. mendica* is found throughout Europe and further east across Iran, Ukraine, Estonia, and Russia, inhabiting suburban areas, woodland, downland and steppe ( GBIF Secretariat, 2022). The species has been recorded across Britain and Ireland, but it is never abundant and is scarce in Scotland ( Randle *et al.*, 2019). The larvae of *D. mendica* feed on a variety of low-growing herbaceous plants. Females will fly in the daytime, around a metre above the ground ( Allan, 1948).

Female *D. mendica* have silky white wings studded with a few black dots. In contrast, most males, including nearly all individuals recorded in Britain, have sooty grey wings with black spots. A polymorphism exists at very low frequency in Britain in which males have creamy white wings, though rarely as pale as in females. This form, common in the south of Ireland and also found in Ukraine, is sometimes given the name var. *rustica* ( Ford, 1967; Khramov, 2023; Onslow, 1921). Crosses suggest the difference is controlled by alleles at a single autosomal locus, with heterozygous males having an intermediate phenotype; other modifier loci also exist ( Ford, 1967; Onslow, 1921). The identity of the major effect gene and the molecular basis of the polymorphism is unknown.

The complete genome of *Diaphora mendica* was determined as part of the Darwin Tree of Life project. The assembled genome will facilitate research into wing patterning and sexual dimorphism in Lepidoptera, and contribute to the growing set of resources for studying insect ecology and evolution.

## Genome sequence report

**Figure 1.**
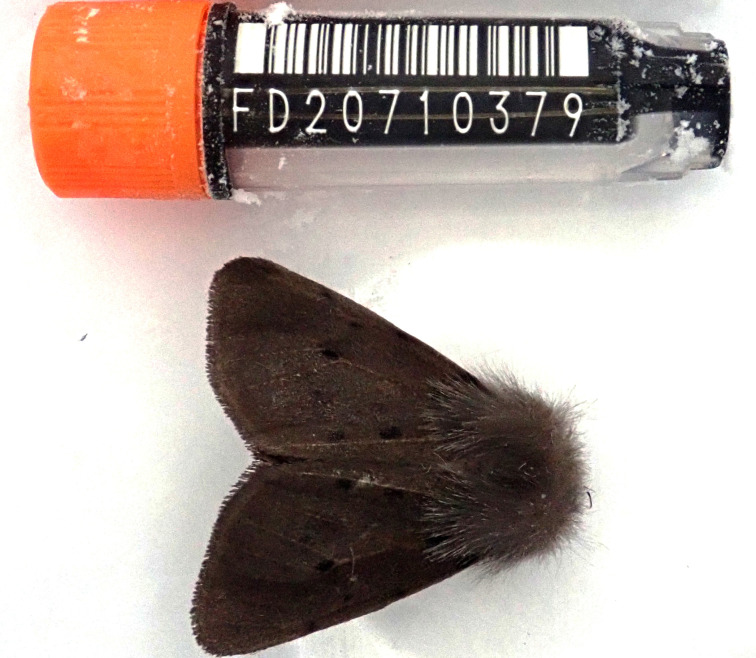
Photograph of the *Diaphora mendica* (ilDiaMend1) specimen used for genome sequencing.

The genome was sequenced from one male *Diaphora mendica* ( Figure 1) collected from Wytham Woods, Oxfordshire, UK (51.77, –1.32). A total of 37-fold coverage in Pacific Biosciences single-molecule HiFi long reads was generated. Primary assembly contigs were scaffolded with chromosome conformation Hi-C data. Manual assembly curation corrected seven missing joins or mis-joins and removed one haplotypic duplication, reducing the scaffold count by 1.

**Figure 2.**
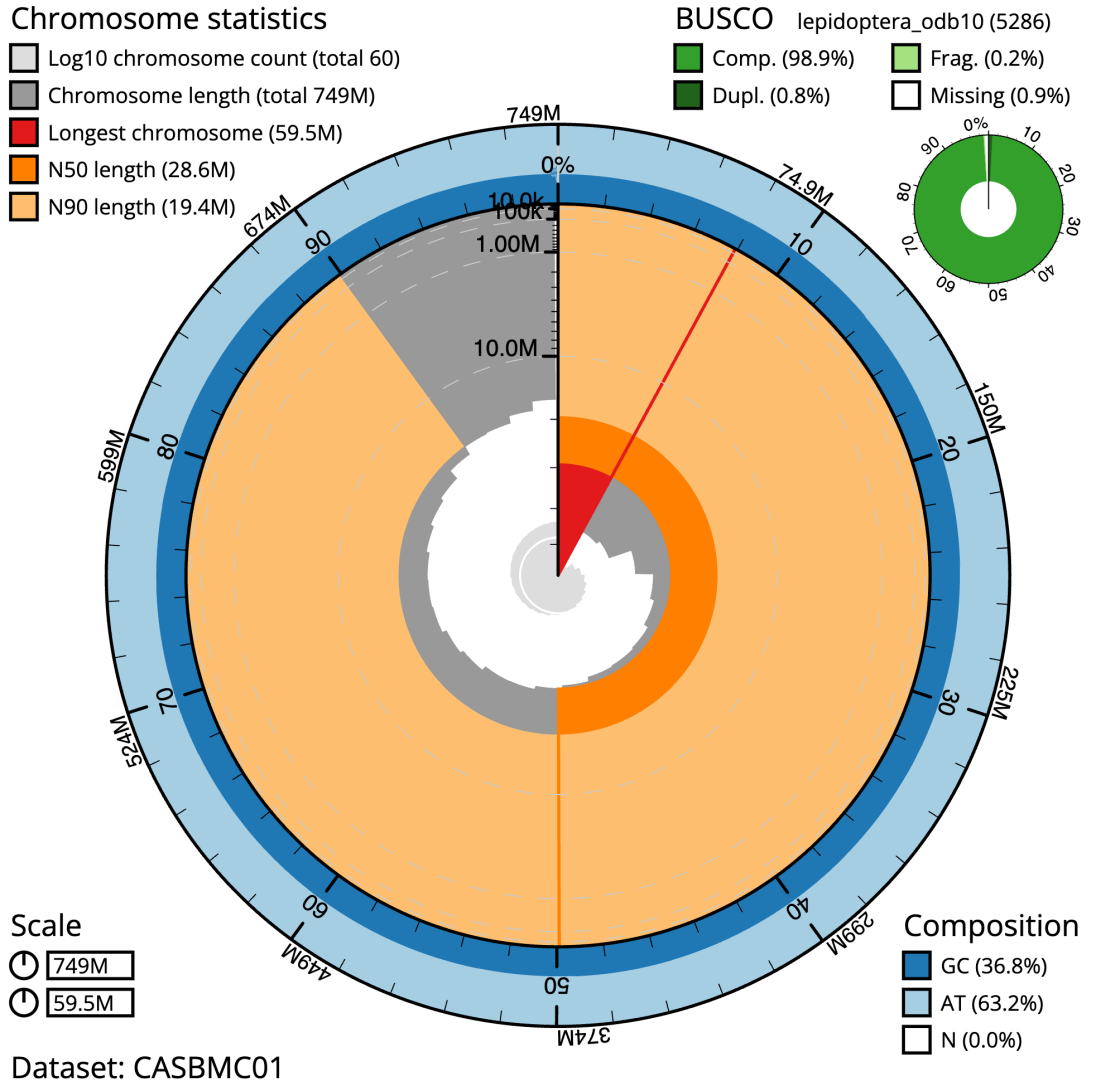
Genome assembly of *Diaphora mendica*, ilDiaMend1.1: metrics. The BlobToolKit Snailplot shows N50 metrics and BUSCO gene completeness. The main plot is divided into 1,000 size-ordered bins around the circumference with each bin representing 0.1% of the 748,682,707 bp assembly. The distribution of scaffold lengths is shown in dark grey with the plot radius scaled to the longest scaffold present in the assembly (59,489,507 bp, shown in red). Orange and pale-orange arcs show the N50 and N90 scaffold lengths (28,641,667 and 19,410,106 bp), respectively. The pale grey spiral shows the cumulative scaffold count on a log scale with white scale lines showing successive orders of magnitude. The blue and pale-blue area around the outside of the plot shows the distribution of GC, AT and N percentages in the same bins as the inner plot. A summary of complete, fragmented, duplicated and missing BUSCO genes in the lepidoptera_odb10 set is shown in the top right. An interactive version of this figure is available at https://blobtoolkit.genomehubs.org/view/ilDiaMend1.1/dataset/CASBMC01/snail.

The final assembly has a total length of 748.7 Mb in 59 sequence scaffolds with a scaffold N50 of 28.6 Mb ( Table 1). Most (99.73%) of the assembly sequence was assigned to 26 chromosomal-level scaffolds, representing 25 autosomes and the Z sex chromosome. Chromosome-scale scaffolds confirmed by the Hi-C data are named in order of size ( Figure 2– Figure 5; Table 2). While not fully phased, the assembly deposited is of one haplotype. Contigs corresponding to the second haplotype have also been deposited. The mitochondrial genome was also assembled and can be found as a contig within the multifasta file of the genome submission.

**Table 1.** Genome data for *Diaphora mendica*, ilDiaMend1.1.

Project accession data		
Assembly identifier	ilDiaMend1.1	
Species	*Diaphora mendica*	
Specimen	ilDiaMend1	
NCBI taxonomy ID	987922	
BioProject	PRJEB58228	
BioSample ID	SAMEA10979149	
Isolate information	ilDiaMend1, male: head and thorax (DNA sequencing and Hi-C scaffolding); abdomen (RNA sequencing)	
Assembly metrics *		*Benchmark*
Consensus quality (QV)	66.9	*≥ 50*
*k*-mer completeness	100%	*≥ 95%*
BUSCO **	C:98.9%[S:98.1%,D:0.8%], F:0.2%,M:0.9%,n:5,286	*C ≥ 95%*
Percentage of assembly mapped to chromosomes	99.73%	*≥ 95%*
Sex chromosomes	Z chromosome	*localised homologous pairs*
Organelles	Mitochondrial genome assembled	*complete single alleles*
Raw data accessions		
PacificBiosciences SEQUEL II	ERR10677842	
Hi-C Illumina	ERR10684068	
PolyA RNA-Seq Illumina	ERR10908615	
Genome assembly		
Assembly accession	GCA_949125395.1	
*Accession of alternate* *haplotype*	GCA_949125405.1	
Span (Mb)	748.7	
Number of contigs	132	
Contig N50 length (Mb)	12.7	
Number of scaffolds	59	
Scaffold N50 length (Mb)	28.6	
Longest scaffold (Mb)	59.5	

* Assembly metric benchmarks are adapted from column VGP-2020 of “Table 1: Proposed standards and metrics for defining genome assembly quality” from ( Rhie *et al.*, 2021). ** BUSCO scores based on the lepidoptera_odb10 BUSCO set using v5.3.2. C = complete [S = single copy, D = duplicated], F = fragmented, M = missing, n = number of orthologues in comparison. A full set of BUSCO scores is available at https://blobtoolkit.genomehubs.org/view/ilDiaMend1.1/dataset/CASBMC01/busco.

**Figure 3.**
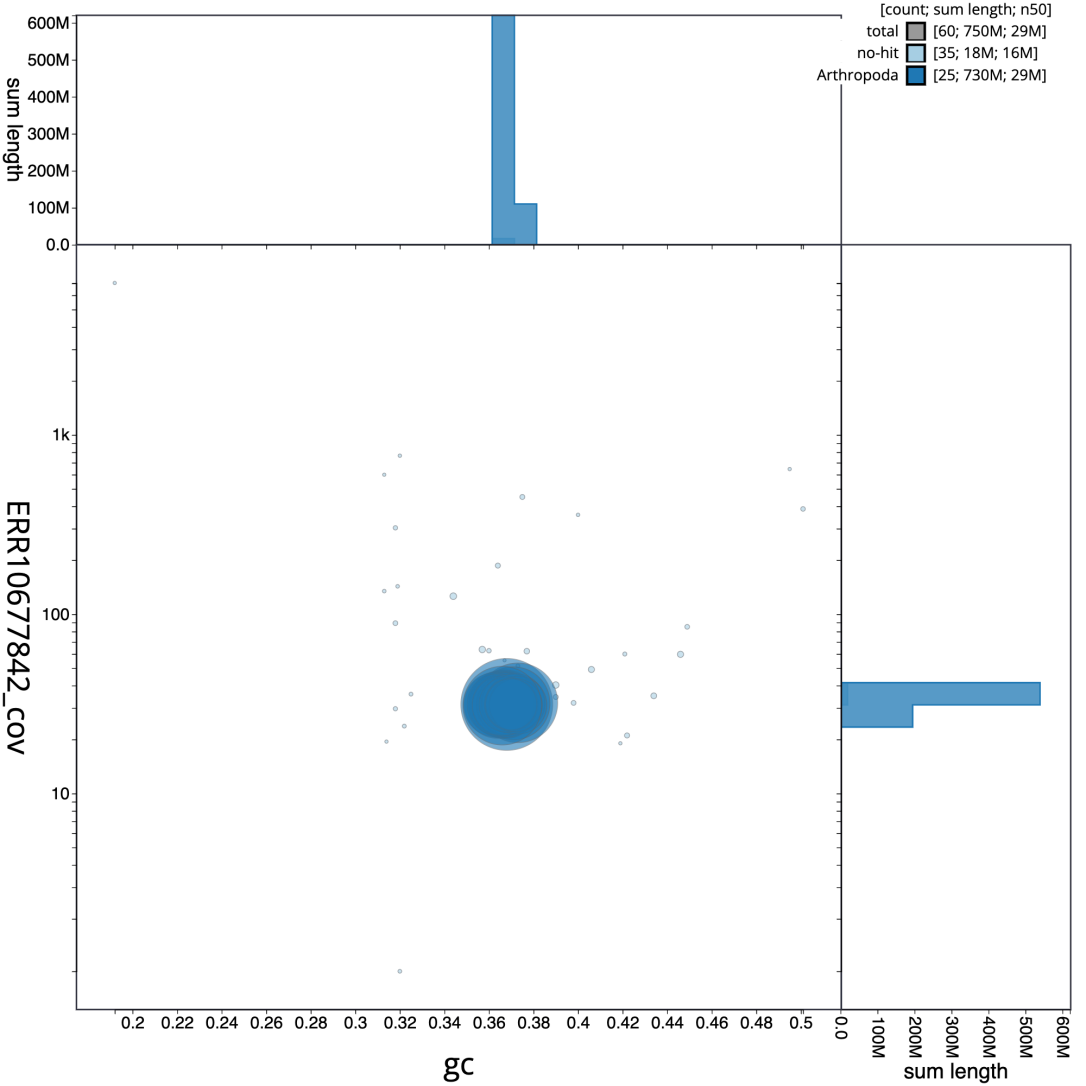
Genome assembly of *Diaphora mendica*, ilDiaMend1.1: BlobToolKit GC-coverage plot. Scaffolds are coloured by phylum. Circles are sized in proportion to scaffold length. Histograms show the distribution of scaffold length sum along each axis. An interactive version of this figure is available at https://blobtoolkit.genomehubs.org/view/ilDiaMend1.1/dataset/CASBMC01/blob.

**Figure 4.**
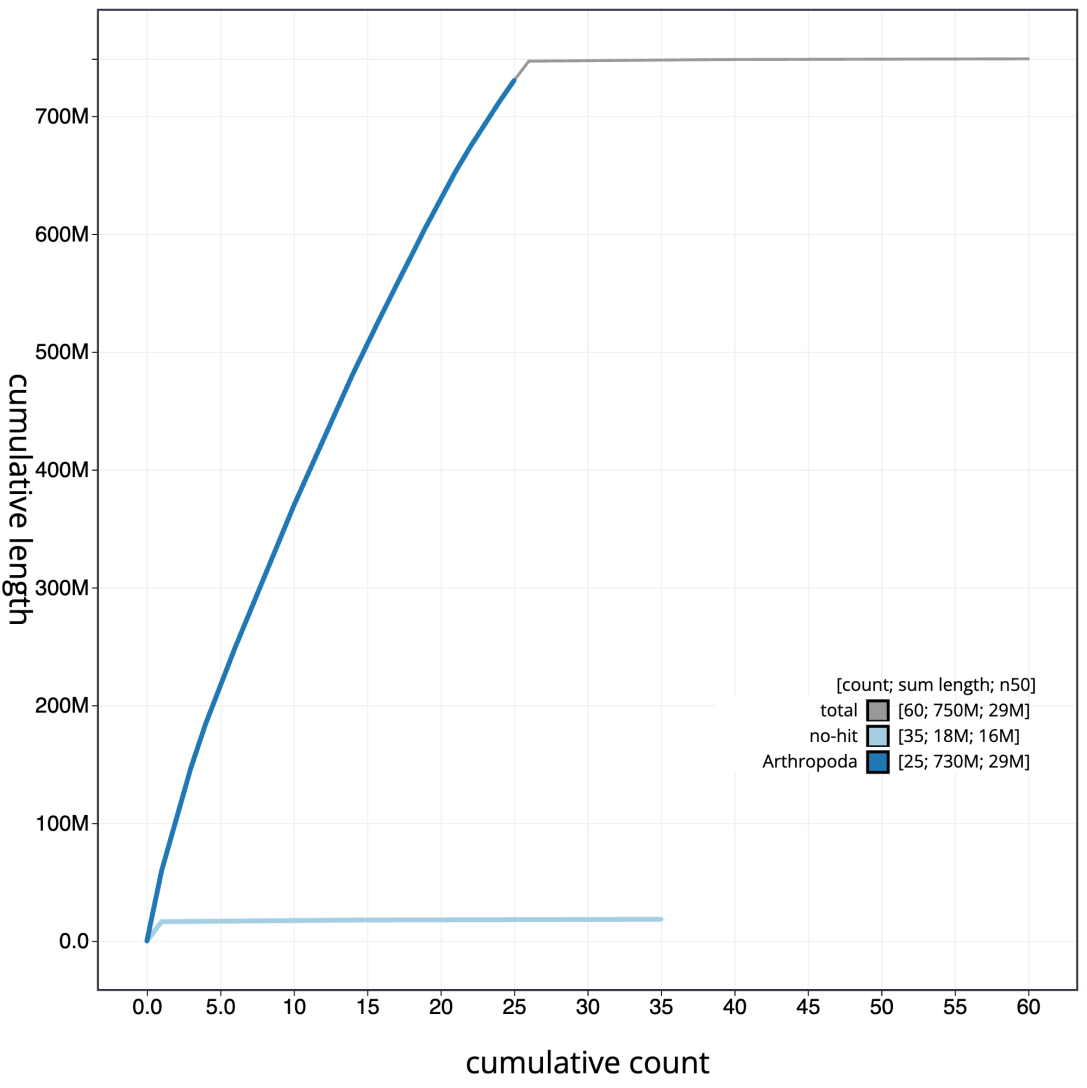
Genome assembly of *Diaphora mendica*, ilDiaMend1.1: BlobToolKit cumulative sequence plot. The grey line shows cumulative length for all scaffolds. Coloured lines show cumulative lengths of scaffolds assigned to each phylum using the buscogenes taxrule. An interactive version of this figure is available at https://blobtoolkit.genomehubs.org/view/ilDiaMend1.1/dataset/CASBMC01/cumulative.

**Figure 5.**
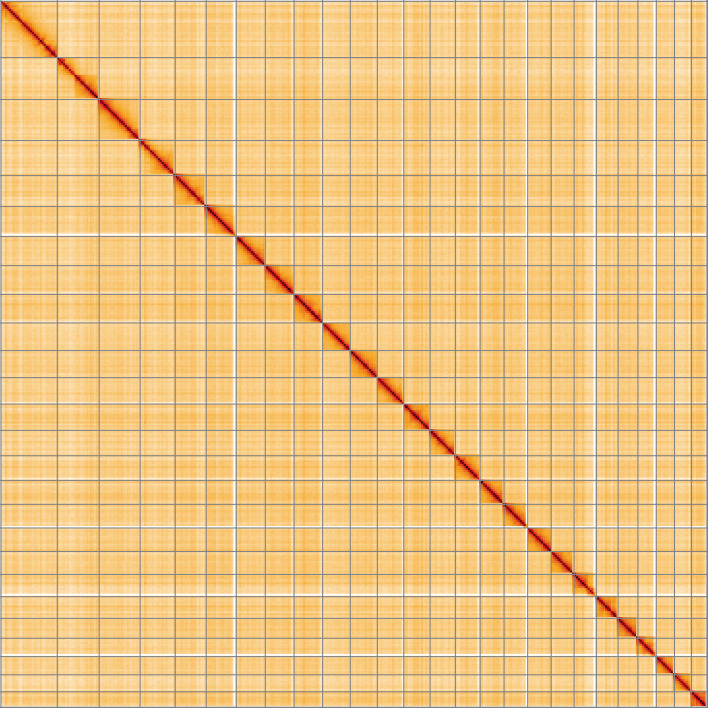
Genome assembly of *Diaphora mendica*, ilDiaMend1.1: Hi-C contact map of the ilDiaMend1.1 assembly, visualised using HiGlass. Chromosomes are shown in order of size from left to right and top to bottom. An interactive version of this figure may be viewed at https://genome-note-higlass.tol.sanger.ac.uk/l/?d=fvxXfR_HSXWL4U_hP21wVQ.

**Table 2.** Chromosomal pseudomolecules in the genome assembly of *Diaphora mendica*, ilDiaMend1.

INSDC accession	Chromosome	Length (Mb)	GC%
OX421310.1	1	59.49	37.0
OX421311.1	2	44.36	37.5
OX421313.1	3	37.12	37.0
OX421314.1	4	32.75	36.5
OX421315.1	5	31.78	36.5
OX421316.1	6	30.74	36.5
OX421317.1	7	30.43	36.5
OX421318.1	8	30.2	36.5
OX421319.1	9	29.25	37.0
OX421320.1	10	28.64	36.5
OX421321.1	11	27.92	36.5
OX421322.1	12	27.86	36.5
OX421323.1	13	26.84	36.5
OX421324.1	14	26.16	36.5
OX421325.1	15	25.22	36.5
OX421326.1	16	25.0	37.0
OX421327.1	17	24.88	36.5
OX421328.1	18	24.24	37.0
OX421329.1	19	23.57	37.0
OX421330.1	20	22.93	37.5
OX421331.1	21	21.01	37.0
OX421332.1	22	19.41	37.0
OX421333.1	23	19.24	37.0
OX421334.1	24	17.95	37.0
OX421335.1	25	16.36	37.0
OX421312.1	Z	43.32	36.5
OX421336.1	MT	0.02	19.5

The estimated Quality Value (QV) of the final assembly is 66.9 with *k*-mer completeness of 100%, and the assembly has a BUSCO v5.3.2 completeness of 98.9% (single = 98.1%, duplicated = 0.8%), using the lepidoptera_odb10 reference set ( *n* = 5,286).

Metadata for specimens, spectral estimates, sequencing runs, contaminants and pre-curation assembly statistics can be found at https://links.tol.sanger.ac.uk/species/987922.

## Methods

### Sample acquisition and nucleic acid extraction

The specimen selected for genome sequencing was a male *Diaphora mendica* (specimen ID Ox001888, individual ilDiaMend1) was collected from Wytham Woods, Oxfordshire (biological vice-county Berkshire), UK (latitude 51.77, longitude –1.32) on 2021-05-28. The specimen was taken from woodland habitat by Douglas Boyes (University of Oxford) using a light trap. The specimen was identified by the collector and preserved on dry ice.

The ilDiaMend1 sample was prepared for DNA extraction at the Tree of Life laboratory, Wellcome Sanger Institute (WSI). The sample was weighed and dissected on dry ice with tissue set aside for Hi-C sequencing. Head and thorax tissue was cryogenically disrupted to a fine powder using a Covaris cryoPREP Automated Dry Pulveriser, receiving multiple impacts. DNA was extracted at the WSI Scientific Operations core using the Qiagen MagAttract HMW DNA kit, according to the manufacturer’s instructions.

RNA was extracted from abdomen tissue of ilDiaMend1 in the Tree of Life Laboratory at the WSI using TRIzol, according to the manufacturer’s instructions. RNA was then eluted in 50 μl RNAse-free water and its concentration assessed using a Nanodrop spectrophotometer and Qubit Fluorometer using the Qubit RNA Broad-Range (BR) Assay kit. Analysis of the integrity of the RNA was done using Agilent RNA 6000 Pico Kit and Eukaryotic Total RNA assay.

### Sequencing

Pacific Biosciences HiFi circular consensus DNA sequencing libraries were constructed according to the manufacturers’ instructions. Poly(A) RNA-Seq libraries were constructed using the NEB Ultra II RNA Library Prep kit. DNA and RNA sequencing was performed by the Scientific Operations core at the WSI on Pacific Biosciences SEQUEL II (HiFi) and Illumina NovaSeq 6000 (RNA-Seq) instruments. Hi-C data were also generated from head and thorax tissue of ilDiaMend1 that had been set aside, using the Arima2 kit and sequenced on the Illumina NovaSeq 6000 instrument.

### Genome assembly, curation and evaluation

Assembly was carried out with Hifiasm ( Cheng *et al.*, 2021) and haplotypic duplication was identified and removed with purge_dups ( Guan *et al.*, 2020). The assembly was then scaffolded with Hi-C data ( Rao *et al.*, 2014) using YaHS ( Zhou *et al.*, 2023). The assembly was checked for contamination and corrected as described previously ( Howe *et al.*, 2021). Manual curation was performed using HiGlass ( Kerpedjiev *et al.*, 2018) and Pretext ( Harry, 2022). The mitochondrial genome was assembled using MitoHiFi ( Uliano-Silva *et al.*, 2022), which runs MitoFinder ( Allio *et al.*, 2020) or MITOS ( Bernt *et al.*, 2013) and uses these annotations to select the final mitochondrial contig and to ensure the general quality of the sequence.

A Hi-C map for the final assembly was produced using bwa-mem2 ( Vasimuddin *et al.*, 2019) in the Cooler file format ( Abdennur & Mirny, 2020). To assess the assembly metrics, the *k*-mer completeness and QV consensus quality values were calculated in Merqury ( Rhie *et al.*, 2020). This work was done using Nextflow ( Di Tommaso *et al.*, 2017) DSL2 pipelines “sanger-tol/readmapping” ( Surana *et al.*, 2023a) and “sanger-tol/genomenote” ( Surana *et al.*, 2023b). The genome was analysed within the BlobToolKit environment ( Challis *et al.*, 2020) and BUSCO scores ( Manni *et al.*, 2021; Simão *et al.*, 2015) were calculated.

Table 3 contains a list of relevant software tool versions and sources.

**Table 3.** Software tools: versions and sources.

Software tool	Version	Source
BlobToolKit	4.0.7	https://github.com/blobtoolkit/blobtoolkit
BUSCO	5.3.2	https://gitlab.com/ezlab/busco
Hifiasm	0.16.1-r375	https://github.com/chhylp123/hifiasm
HiGlass	1.11.6	https://github.com/higlass/higlass
Merqury	MerquryFK	https://github.com/thegenemyers/MERQURY.FK
MitoHiFi	2	https://github.com/marcelauliano/MitoHiFi
PretextView	0.2	https://github.com/wtsi-hpag/PretextView
purge_dups	1.2.3	https://github.com/dfguan/purge_dups
sanger-tol/genomenote	v1.0	https://github.com/sanger-tol/genomenote
sanger-tol/readmapping	1.1.0	https://github.com/sanger-tol/readmapping/tree/1.1.0
YaHS	1.2a	https://github.com/c-zhou/yahs

### Wellcome Sanger Institute – Legal and Governance

The materials that have contributed to this genome note have been supplied by a Darwin Tree of Life Partner. The submission of materials by a Darwin Tree of Life Partner is subject to the **‘Darwin Tree of Life Project Sampling Code of Practice’**, which can be found in full on the Darwin Tree of Life website here. By agreeing with and signing up to the Sampling Code of Practice, the Darwin Tree of Life Partner agrees they will meet the legal and ethical requirements and standards set out within this document in respect of all samples acquired for, and supplied to, the Darwin Tree of Life Project.

Further, the Wellcome Sanger Institute employs a process whereby due diligence is carried out proportionate to the nature of the materials themselves, and the circumstances under which they have been/are to be collected and provided for use. The purpose of this is to address and mitigate any potential legal and/or ethical implications of receipt and use of the materials as part of the research project, and to ensure that in doing so we align with best practice wherever possible. The overarching areas of consideration are:

- Ethical review of provenance and sourcing of the material
- Legality of collection, transfer and use (national and international)

Each transfer of samples is further undertaken according to a Research Collaboration Agreement or Material Transfer Agreement entered into by the Darwin Tree of Life Partner, Genome Research Limited (operating as the Wellcome Sanger Institute), and in some circumstances other Darwin Tree of Life collaborators.

## Data Availability

European Nucleotide Archive: *Diaphora mendica* (muslin). Accession number PRJEB58228; https://identifiers.org/ena.embl/PRJEB58228. ( Wellcome Sanger Institute, 2023) The genome sequence is released openly for reuse. The *Diaphora mendica* genome sequencing initiative is part of the Darwin Tree of Life (DToL) project. All raw sequence data and the assembly have been deposited in INSDC databases. The genome will be annotated using available RNA-Seq data and presented through the Ensembl pipeline at the European Bioinformatics Institute. Raw data and assembly accession identifiers are reported in Table 1.

## Author information

Members of the University of Oxford and Wytham Woods Genome Acquisition Lab are listed here: https://doi.org/10.5281/zenodo.4789928.

Members of the Darwin Tree of Life Barcoding collective are listed here: https://doi.org/10.5281/zenodo.4893703.

Members of the Wellcome Sanger Institute Tree of Life programme are listed here: https://doi.org/10.5281/zenodo.4783585.

Members of Wellcome Sanger Institute Scientific Operations: DNA Pipelines collective are listed here: https://doi.org/10.5281/zenodo.4790455.

Members of the Tree of Life Core Informatics collective are listed here: https://doi.org/10.5281/zenodo.5013541.

Members of the Darwin Tree of Life Consortium are listed here: https://doi.org/10.5281/zenodo.4783558.
