# Supplementary material for: The genome sequence of the Muslin moth, Diaphora mendica (Clerck, 1759)
Source: Wellcome Open Res. 2023 Jun 21;8:257. [Version 1] doi: 10.12688/wellcomeopenres.19540.1 (PMC10806365; doi:10.12688/wellcomeopenres.19540.1)
Supplement: Peer review history

# Reviewer report: Reviewer response for version 1

**DOI:** 10.21956/wellcomeopenres.21644.r71871

**Published:** 2024-01-23

**Authors:** Annabel Whibley

**Affiliations:** 1 The University of Auckland, Auckland, Auckland, New Zealand; 2 Grapevine Improvement, Bragato Research Institute, LIncoln, New Zealand

This genome note describes the sequencing and genome assembly of *Diaphora medica* (the Muslin moth) under the auspices of the Darwin Tree of Life Consortium. The sample collection, sequencing, metadata and assembly methods used are described concisely, accurately and comprehensively and are available for use. The moth is of particular interest in respect to sexual dimorphism (and male polymorphism) in wing patterning, and this assembly provides a useful foundation for further study. The assembly quality is extremely high when set against VGP metrics for a primary assembly.

 Although RNAseq data was obtained, the annotation is pending. The annotation will add further value to the dataset.

Are sufficient details of methods and materials provided to allow replication by others?

Yes

Is the rationale for creating the dataset(s) clearly described?

Yes

Are the datasets clearly presented in a useable and accessible format?

Yes

Are the protocols appropriate and is the work technically sound?

Yes

Reviewer Expertise:

Genomics, Evolution

I confirm that I have read this submission and believe that I have an appropriate level of expertise to confirm that it is of an acceptable scientific standard.

# Reviewer report: Reviewer response for version 1

**DOI:** 10.21956/wellcomeopenres.21644.r68230

**Published:** 2023-10-13

**Authors:** Xiangyu Hao

**Affiliations:** 1 College of Plant Protection, Northwest A&F University, Yangling, Shaanxi, China

The manuscript presents a comprehensive genome assembly of the Muslin moth, *Diaphora mendica*, and provides potentially valuable insights into the genetic basis of sexual dimorphism and wing patterning in Lepidoptera. The study demonstrates a meticulous approach to genome sequencing and assembly, resulting in a high-quality reference genome for this species. The research is well-documented, and the methods are sound, contributing to our understanding of evolutionary biology and insect ecology. I recommend accepting this manuscript for indexing.

Are sufficient details of methods and materials provided to allow replication by others?

Yes

Is the rationale for creating the dataset(s) clearly described?

Yes

Are the datasets clearly presented in a useable and accessible format?

Yes

Are the protocols appropriate and is the work technically sound?

Yes

Reviewer Expertise:

Evolutionary Genomics, Entomology, Systematics

I confirm that I have read this submission and believe that I have an appropriate level of expertise to confirm that it is of an acceptable scientific standard.
